# Attitudes toward a COVID-19 vaccine and vaccination status in cancer patients: a cross-sectional survey

**DOI:** 10.1007/s00432-022-03961-y

**Published:** 2022-02-26

**Authors:** Svenja Heyne, Peter Esser, Anne Werner, Antje Lehmann-Laue, Anja Mehnert-Theuerkauf

**Affiliations:** Department of Medical Psychology and Medical Sociology, University Medical Center Leipzig, Leipzig, Germany

**Keywords:** Anxiety, Cancer, COVID-19, Hesitancy, Oncology, Vaccination

## Abstract

**Purpose:**

We aim to assess attitudes toward a COVID-19 vaccine and vaccination status in cancer patients and to explore additional factors such as the level of information and comprehensibility and accessibility of this information, anxiety symptoms in general and toward COVID-19, and general health literacy.

**Methods:**

We included 425 outpatients (mean age 61.4, age range 30–88 years, 60.5% women) of the Psychosocial Counseling Center for Cancer patients of the Department of Medical Psychology and Medical Sociology, Leipzig. We recorded attitudes toward a COVID-19 vaccine and vaccination status via self-report. The impact of psychosocial factors, including anxiety (GAD-7), COVID-19-specific anxiety (OCS; FCV-19S) and health literacy (HLS-EU-Q16) were analyzed with point-biserial correlations using Pearson’s r.

**Results:**

We found that the vast majority (95.5%) reported being vaccinated against COVID-19 and that overall trust in safety and protective effects of a COVID-19 vaccine was high (90.9%). The vaccination readiness among nonvaccinated cancer survivors was low to very low with “fear of side effects” the most mentioned (72.2%) reason against a COVID-19 vaccine. There was no significant correlation between vaccination status and fear or anxiety symptomatology, and health literacy. Obsessive thoughts about COVID-19 was significantly higher in nonvaccinated cancer patients.

**Conclusions:**

Majority of respondents are positive about COVID-19 vaccine, accompanied by a very high rate of COVID-19 immunization in our sample. Further studies with a larger sample of nonvaccinated cancer patients should further investigate the relationship on fear and vaccination hesitancy and align communication strategies accordingly.

## Introduction

Numerous studies have shown that patients with cancer are at increased risk of severe or fatal outcome with COVID-19 (Barbui et al 2021; Chari et al 2020; Desai et al 2020, 2021; Kuderer et al 2020; Richardson et al 2020; Sharma et al 2021; Subbiah 2020). In a study from the UK, mortality in hospitalized patients with cancer was 40.5% versus 28.5% (HR 1.62; *p* < 0.001) in non-cancer patients (Palmieri et al 2020). In the German LEOSS registry, mortality in cancer patients was 22.5% versus 14% (*p* < 0.001) in non-cancer patients (Rüthrich et al 2021). In addition to the implementation of general hygiene and infection control measures, the use of vaccines against COVID-19 has a central role in combating the pandemic (Harder et al 2021). Vaccines against COVID-19 elicit a protective immune response and are critical in preventing and reducing the morbidity and mortality rates caused by SARS-CoV-2 infections (Poland et al 2020). International efforts in the development and licensure of several vaccines led to the start of vaccination campaigns as early as one year after the onset of the pandemic. Efficacy in more than 90% of subjects and a good safety profile of the vaccines have been demonstrated in studies (Baden et al 2021; Polack et al 2020; Voysey et al 2021). Current data indicate that most people have strong protection against serious illness and death for at least 6 months after their second dose. Immunity may reduce faster in people who are older or who have underlying medical conditions, like people who are moderately or severely immunocompromised due to their cancer treatment (NCI 2021).

Because of former limited vaccine resources, groups of persons were vaccinated against SARS-CoV-2 in Germany with different prioritization (BMJ 2021). On June 7, 2021, the German government lifted vaccination prioritization. All people in Germany over the age of 12 could then generally get a vaccination appointment. However, in many countries, hesitation and the deliberate dissemination of misinformation or incomplete information are significant barriers to achieve widespread immunization of the population (Machingaidze and Wiysonge 2021; Cornwall 2020). As previous study results suggest, factors influencing COVID-19 vaccination acceptance and hesitancy among cancer patients are concern with worsening the prognosis of the cancer treatment, critical evaluation of vaccines’ efficacy and safety, and knowledge on the COVID-19 vaccination process (Brodziak et al 2021, Chun et al. 2021, Hong et al 2022).

Given the lack of data on COVID-19 vaccination among cancer patients in Germany, the aim of our observational study was to determine attitudes and vaccination status with a COVID-19 vaccine among (former) cancer patients of different cancer sites. We recorded attitudes toward a COVID-19 vaccine and vaccination status. In addition, we assessed potential associated factors such as the level of information, comprehensibility and accessibility of this information, anxiety symptoms in general and toward COVID-19, and general health literacy.

## Methods

### Study design and sample

The population of this cross-sectional study includes outpatients of the Psychosocial Counseling Center for Cancer patients of the Department of Medical Psychology and Medical Sociology, University Medical Center Leipzig, who had previously agreed to be contacted regarding scientific studies.

Patients were eligible for study participation if they had a confirmed diagnosis of cancer, were at least 18 years of age at the time of diagnosis and who were fluent in written and spoken German. All participants gave written informed consent in accordance with the Declaration of Helsinki. The study was approved by the Research Ethics Committee of the University of Leipzig (Ref. 266/21-ek).

## Study recruitment and data collection

Eligible patients received a study information letter, together with a letter of consent and the paper and pencil-based questionnaire by mail. Using a prepaid envelope, patients could return the completed questionnaire including informed consent. A response form was included, in case patients did not wish to participate. Patients were able to indicate their reason(s) for non-participation and sent it back to the study team.

## Study measures

### COVID-19 disease history and immunization

COVID-19 immunization was covered by questions on vaccination status, number of vaccination(s) received. In addition, perceived reasons for or against COVID-19 vaccination, and questions about disease history regarding COVID-19 infection in oneself or a close relative was recorded via self-generated questions.

### Attitude toward a (COVID-19) vaccination

We used a self-generated nine-item scale with statements about COVID-19 vaccination and vaccinations in general. Respondents were asked to indicate their agreement on a five-point Likert scale ranging from 1 “strongly disagree” to 5 “strongly agree”. Higher scores indicate greater confidence in COVID-19 vaccination and vaccinations in general, as well as in the relevant parties making recommendations in this regard.

### Level, comprehensibility and accessibility of information on COVID-19 vaccination

We inquired about the source(s) from which respondents obtained their information regarding COVID-19 vaccination. In addition, we used a self-generated scale where respondents indicate their agreement or disagreement on items such as the level of information, comprehensibility and accessibility of this information. Items were rated on a five-point Likert scale ranging from 1 “strongly disagree” to 5 “strongly agree”, with higher scores indicating higher agreement.

### Fear and anxiety symptomatology

We assessed General anxiety disorder symptomatology using the validated German version of the Generalized Anxiety Disorder Scale (GAD-7; Spitzer et al 2006). The GAD-7 has a high internal consistency (Cronbach’s α > 0.83). The frequency of symptoms within the last two weeks is rated on a four‐point Likert scale ranging from 0 “not at all” to 3 “nearly every day”. The sum score of the GAD‐7 ranges from 0 to 21, with values of 0–4, 5–9, 10–14, and 15–21 indicating minimal, mild, moderate, or severe anxiety symptoms (Spitzer et al 2006).

We measured obsessive thinking about COVID-19 using the Obsession with COVID-19 Scale (OCS; Lee 2020). The OCS is a self-report mental health screener of persistent and disturbed thinking about COVID-19 with a good internal consistency (Cronbach’s α > 0.83). The four items are rated on a five-point Likert scale ranging from 0 “not at all” to 4 “nearly every day”, based on experiences over the past two weeks. Elevated scores on a particular item or a high total score (≥ 7) may indicate problematic symptoms and probable dysfunctional thoughts about COVID-19 (Lee 2020).

We assessed anxiety toward COVID-19 using the Fear of COVID-19 Scale (FCV-19S; Ahorsu et al 2020). The FCV-19S assesses fears emanating from COVID-19 with a good internal consistency (Cronbach’s α > 0.82). On seven items the participants indicate their level of agreement on a five-point Likert scale ranging from 1 “strongly disagree” to 5 “strongly agree”. A total score is calculated by adding up each item score (ranging from 7 to 35). Higher scores indicate greater fear of COVID-19 (Ahorsu et al 2020).

The FCV-19S and the OCS were both translated according to the recommendations of the International Testing Commission (ITC; ITC 2017). The forward–backward translation method was used to translate the FCV-19S and the OCS into German. Two independent translators (PE and SH) translated the Fear of COVID-19 scale into German. Both versions were then translated back into English by a professional translator with experience in psychological research. The original and back-translated versions were then compared and differences were discussed. This went on until the two reviewers considered both versions to be equivalent.

### Health literacy

Health literacy was assessed using the German version of the European Health Literacy Survey Questionnaire (HLS-EU-Q16, Jordan and Hoebel 2015). The HLS-EU-Q16 is clustered in four dimensions (“access”, “understand”, “appraise” and “apply” health information) and three different domains (“health care”, “disease prevention”, and “health promotion”). The 16 items are scored on a four-point Likert scale ranging from 1 “very difficult” to 4 “very easy” with higher scores indicating better health literacy (Sørensen et al. 2012).

## Statistical analysis

We applied descriptive analyses for both continuous (frequencies, mean, and standard deviation) and categorical variables (frequencies, percentages).

Comparisons between participants and non-responders were performed with t-test for the continuous variable age and Chi-square test for the categorical variable gender. Point-biserial correlations using Pearson’s r was performed to calculate the relationship between vaccination status and anxiety symptoms and health literacy.

Due to missing reference data and a better interpretability, the scales “attitude toward (COVID-19) vaccination” and “level, comprehensibility and accessibility of information on COVID-19 vaccination” were recoded. Response categories 1 “strongly disagree” and 2 “disagree” were combined into one category coded as 1 “disagreement”, 3 “neither nor” into one category coded as 2 “neither nor”, 4 “agree” and 5 “strongly agree” into one category coded as 3 “agreement”. Response categories from the Health Literacy scale 1 “very easy” and 2 “fairly easy” were merged into one category coded as “1”, 3 “fairly difficult” and 4 “very difficult” into another category coded as “0”. The sum score is ranging from 0 to 16. Patients with at least 11 answers were considered in the final analysis. We then calculated the mean, multiplied it with 16 and clustered patients into the following categories: inadequate (0–8 points), problematic (> 8–12 points) and sufficient health literacy (> 12–16 points), subsuming the first two categories into *limited* health literacy (Pelikan et al 2013).

In all analyses, two-sided p < 0.05 were considered significant. Data analyses were performed with IBM SPSS Statistics 27 (IBM Corp., 2020).

## Results

### Sample

**Fig. 1:**
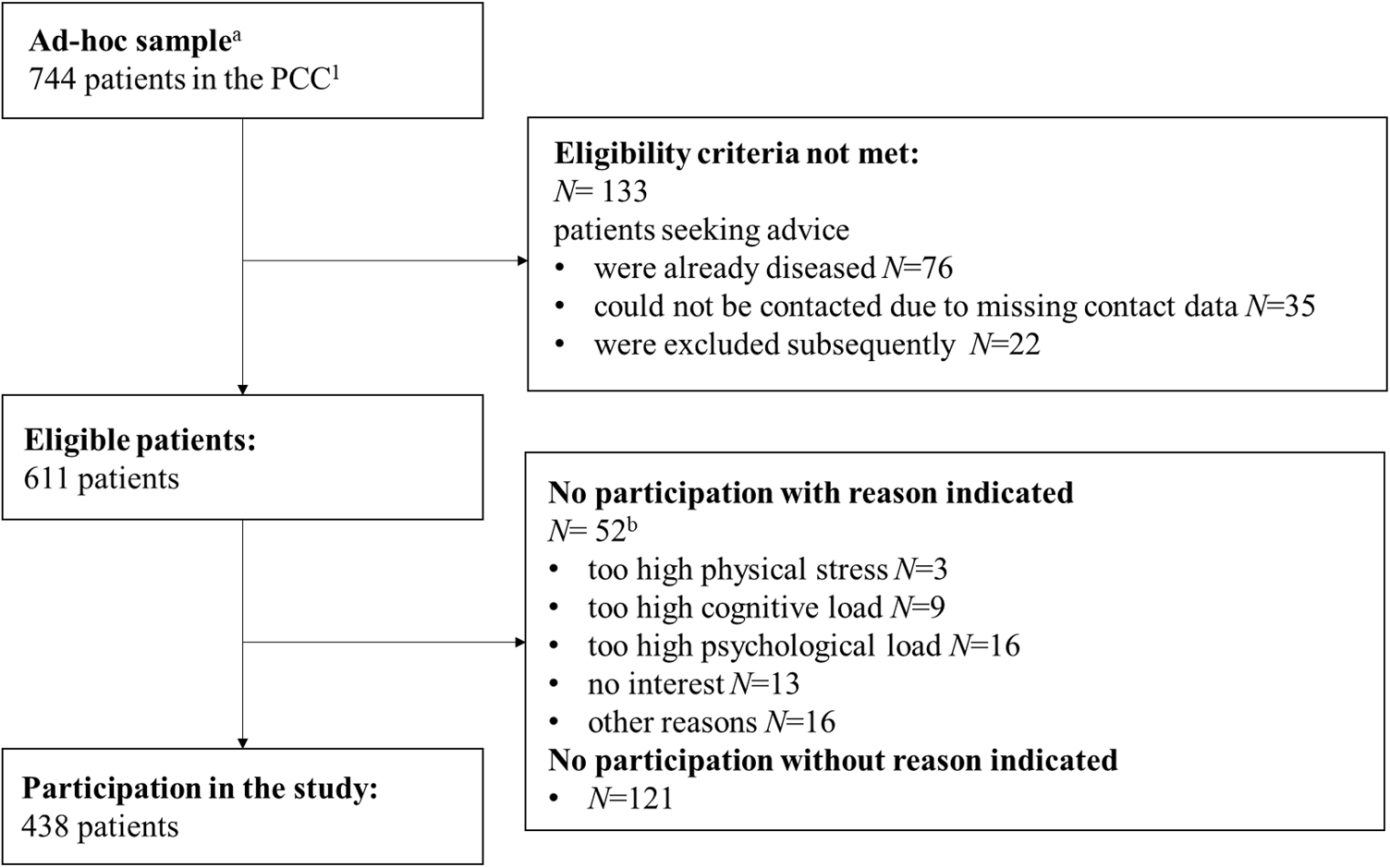
Flow-chart of participants. ^**1**^Psychosocial Counseling Center for Cancer patients, ^a^patients seeking advice in the years 2017/18/19/20, ^b^possibility for multiple answers was given

Patient recruitment was carried out from 2021 September to 2021 November. Out of 744 eligible patients, 438 (response rate: 59.0%) participated in the study (Fig. 1). Among those, 425 patients returned a complete questionnaire and a letter of consent and were included in the final analysis.

Mean age was 61.4 years (*SD* = 12.3) and 60.5% were female. Breast cancer was the most frequent cancer diagnosis (38.6%), and respondents’ median time since diagnosis was 4 years (*IQR* 3 to 5 years). The majority of patients (67.5%) were not under treatment. Study participants were older than non-responders (*p* < 0.001) with no significant differences in gender (*p* = 0.307). Table 1 shows social and medical characteristics for the total sample.

**Table 1:** Characteristics for participants

	Total sample
	*N* (%)
	425 (100.0)
Sociodemographic data	
Sex	
Male	168 (39.5)
Female	257 (60.5)
Age in years, M (SD)	61.4 (12.3)
30–39	20 (4.7)
40–50	59 (13.9)
51–60	126 (29.6)
61–70	113 (26.6)
71–80	85 (20.0)
> 80	22 (5.2)
Marital status^1^	
Single	64 (15.2)
Married	273 (64.7)
Divorced	51 (12.1)
Living apart	5 (1.2)
Widowed	29 (6.9)
Professional education^2^	
Apprenticeship	168 (40.5)
Technical college, engineering school	81 (19.5)
University, college	133 (32.0)
Other	30 (7.2)
Without vocational training	3 (0.7)
Clinical data	
Cancer diagnosis	
Breast	162 (38.6)
Male genital organs	81 (19.1)
Hematological	43 (10.1)
Digestive organs	38 (9.0)
Skin	29 (6.8)
Lip, oral cavity and pharynx	24 (5.6)
Female genital organs	23 (5.4)
Respiratory organs	18 (4.3)
Eye, brain and other parts of central nervous system	16 (3.8)
Urinary tract	16 (3.7)
Other^a^	14 (3.3)
Cancer-related data	
Second cancer disease	35 (8.2)
Time since cancer diagnosis^b,3^, Mdn (IQR)	3.98 (2.08)
1st year after diagnosis	12 (2.9)
2nd–3rd year after diagnosis	148 (35.4)
4th year and ongoing	258 (60.7)
Cancer therapy status^4^	
Not under treatment	276 (67.5)
Under treatment	127 (31.1)
Treatment planned	6 (1.5)
Received treatments^c,5^	
Surgery	341 (80.4)
Radiotherapy	318 (75.0)
Chemotherapy	157 (37.0)
Immune therapy	50 (11.8)
Hormone therapy	45 (10.6)
Other	28 (6.2)

^1^n/a = 3

^2^n/a = 10

^3^n/a = 7

^4^n/a = 16

^5^n/a = 1

^a^ Including diagnosis of “thyroid and other endocrine glands”, “bone and articular cartilage”, “mesothelial and soft tissue”

^b^Categories were formed in accordance to Mullan ( 1985), Powel and Seibert (2017), and Rowland et al. (2013)

^c^Possibility of multiple answers was given

### COVID-19 disease history and immunization

Of 425 participants, 406 (95.5%) received a vaccination against SARS-CoV-2. The majority (90.4%) is double-vaxed with the vaccine Comirnaty® (BioNTech/Pfizer, 70.9%). Table 2 shows absolute and percentage frequencies on COVID-19 and immunization-specific characteristics in the two groups “vaccinated” and “nonvaccinated”.

**Table 2:** Frequencies on COVID-19 and immunization-specific characteristics

	Vaccinated		Nonvaccinated	
	*N*	(%)	*N*	(%)
Total	406	(95.5)	19	(4.5)
Ever had COVID-19 before	20	(4.9)	4	(22.2)
Relatives had COVID-19 before	87	(21.5)	3	(15.8)
Vaccination willingness				
Very low			9	(50.0)
Low			1	(5.6)
Undecided			5	(27.8)
High			3	(16.7)
Vaccination received				
Once	20	(4.7)		
Twice	384	(90.4)		
Three times	2	(0.5)		
Vaccine^1,a^				
Comirnaty® (BioNTech/Pfizer)	322	(79.5)		
Vaxzevria® (AstraZeneca)	87	(21.5)		
Spikevax® (Moderna)	26	(6.4)		
COVID-19 Vaccine Janssen	7	(1.6)		

^1^n/a = 1

^a^the possibility of multiple answers was given due to possibility of heterologous vaccination scheme

### Perceived reason(s) for or against COVID-19 vaccination

**Fig. 2:**
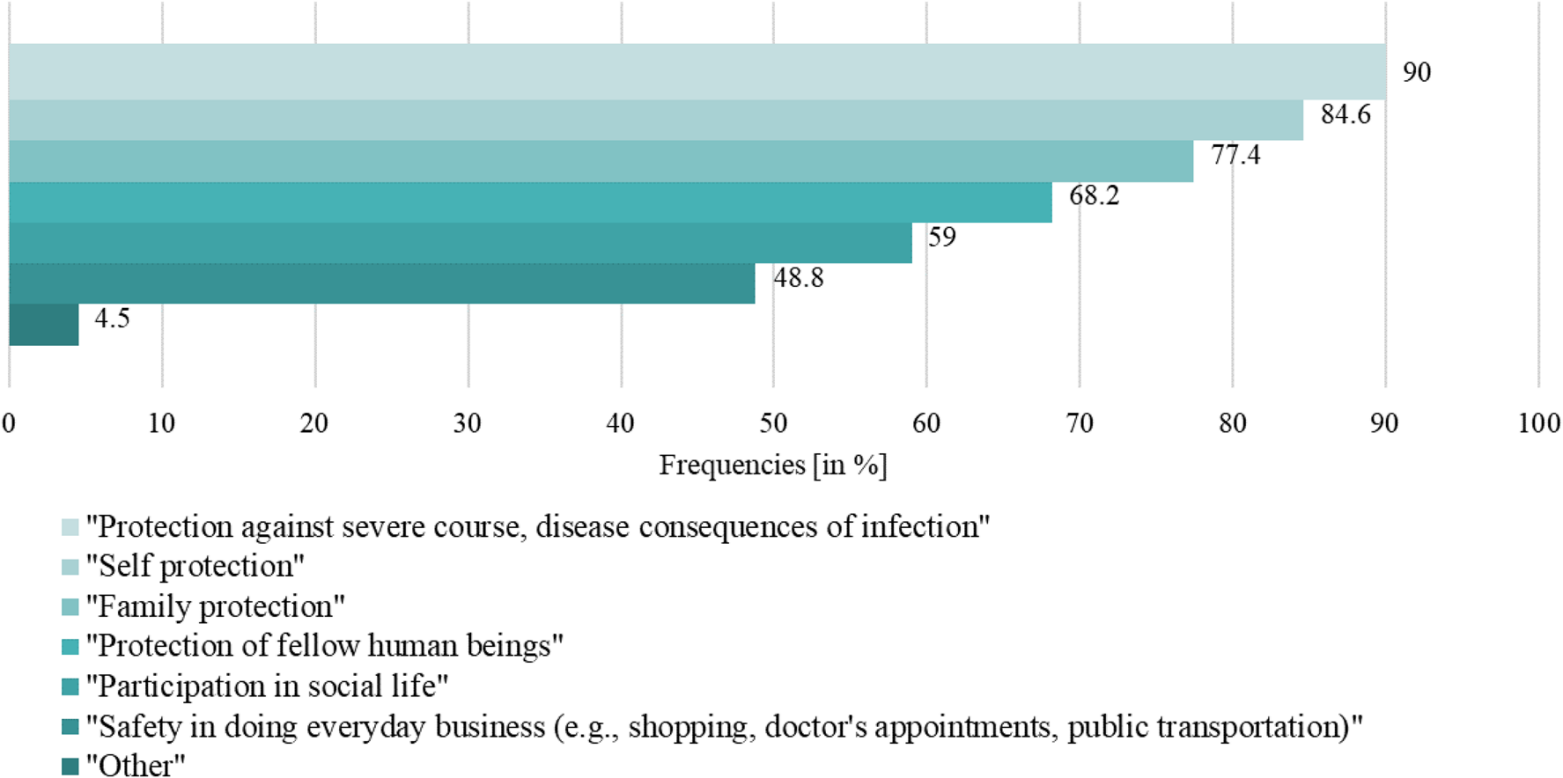
Proportion of reason(s) for vaccination against COVID-19 (*N* = 402^1^). ^1^The possibility of multiple answers was given

Most vaccinated respondents indicated four or more reasons for COVID-19 vaccination (65.6%). Figure 2 gives a detailed overview on the reasons mentioned. “Protection against severe course, disease consequences of infection” was the most mentioned (90.0%) reason, compared to the least mentioned (48.8%) reason “safety in everyday business (e.g., shopping, doctor’s appointments, public transport)”.

**Fig. 3:**
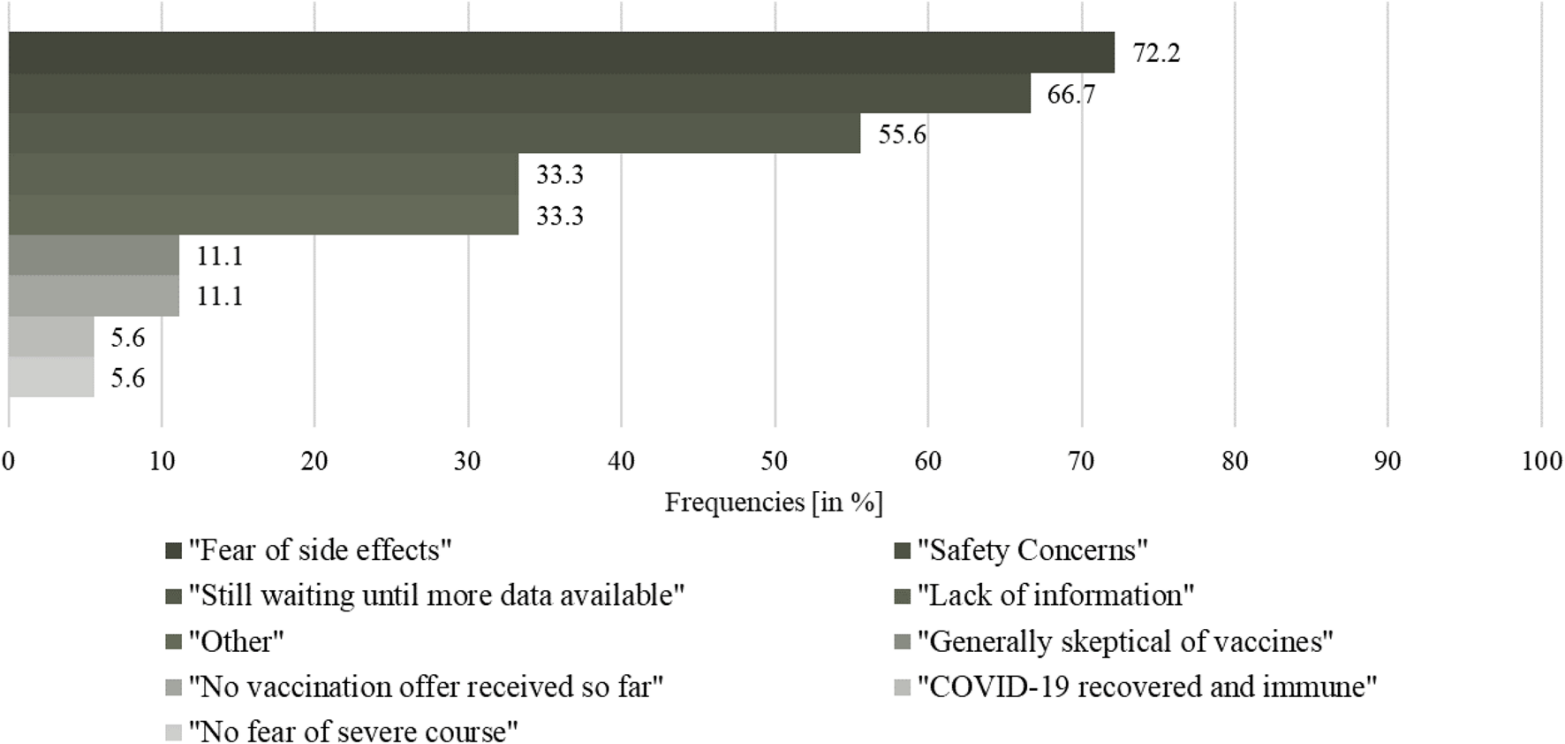
Proportion of reason(s) against vaccination against COVID-19 (*N* = 18^1^). ^1^The possibility of multiple answers was given

The majority (97.2%) of nonvaccinated respondents indicated more than two reasons against a COVID-19 vaccination. Figure 3 gives an overview on the reasons mentioned. “Fear of side effects” was the most mentioned (72.2%) reason, “COVID-19 recovered and immune” and “no fear of severe course” were both the least mentioned (5.6%) reasons.

### Attitude toward a COVID-19 vaccination and vaccination in general

The vast majority of respondents indicate that they “believe a vaccination against COVID-19 reduces the risk of contracting COVID-19.” (90.9%) and that “vaccinations protect against contagious diseases.” (93.8%). The most trusted sources of information on COVID-19 vaccines are expert groups, e.g., Ständige Impfkommission (STIKO) and treating physicians with both 85.2%. Table 3 shows the percentage of agreement and disagreement to each item on trustworthiness of information, belief in protective efficacy, and personal attitudes toward COVID-19 vaccination for the total sample.

**Table 3:** Percentage of agreement and disagreement with statements regarding attitudes toward a (COVID-19) vaccination

	Level of expression		
	Disagreement	Neither nor	Agreement
	*n* (%)	*n* (%)	*n* (%)
Trustworthiness of information on vaccination			
“I trust the information (on safety, risks and side effects, etc.) provided by the government regarding the vaccines against COVID-19.”^1^	51 (13.2)	67 (17.4)	268 (69.4)
“I trust the expert groups (e.g., STIKO) regarding the safety monitoring of the vaccines against COVID-19.”^2^	30 (7.6)	28 (7.1)	335 (85.2)
“I trust the statements of my attending physician regarding the vaccines against COVID-19.”^3^	11 (2.9)	45 (11.9)	322 (85.2)
Belief in protective effects of vaccination			
“I believe that vaccination against COVID-19 reduces the risk of contracting COVID-19.”^4^	17 (4.3)	19 (4.8)	361 (90.9)
“Vaccinations protect against contagious diseases.”^5^	6 (1.6)	18 (4.7)	360 (93.8)
“Vaccinations cause diseases/illnesses.”^6^	270 (73.0)	73 (19.7)	27 (7.3)
Personal attitude toward vaccination			
“It should be up to each person to get vaccinated against COVID-19.”^7^	177 (47.3)	55 (14.7)	142 (38.0)
“I had/have no preference regarding a particular vaccine against COVID-19.”^8^	157 (4.3)	68 (18.3)	146 (39.4)
“I will get/have gotten vaccinated as soon as a COVID-19 vaccine is/was available to me.”^9^	24 (6.6)	15 (4.1)	326 (89.3)

^*1*^n/a = 39

^*2*^n/a = 32

^*3*^n/a = 47

^*4*^n/a = 28

^*5*^n/a = 51

^*6*^n/a = 54

^*7*^n/a = 60

^*8*^n/a = 41

^*9*^n/a = 55

### Level, comprehensibility, and accessibility of information on COVID-19 vaccination

**Fig. 4:**
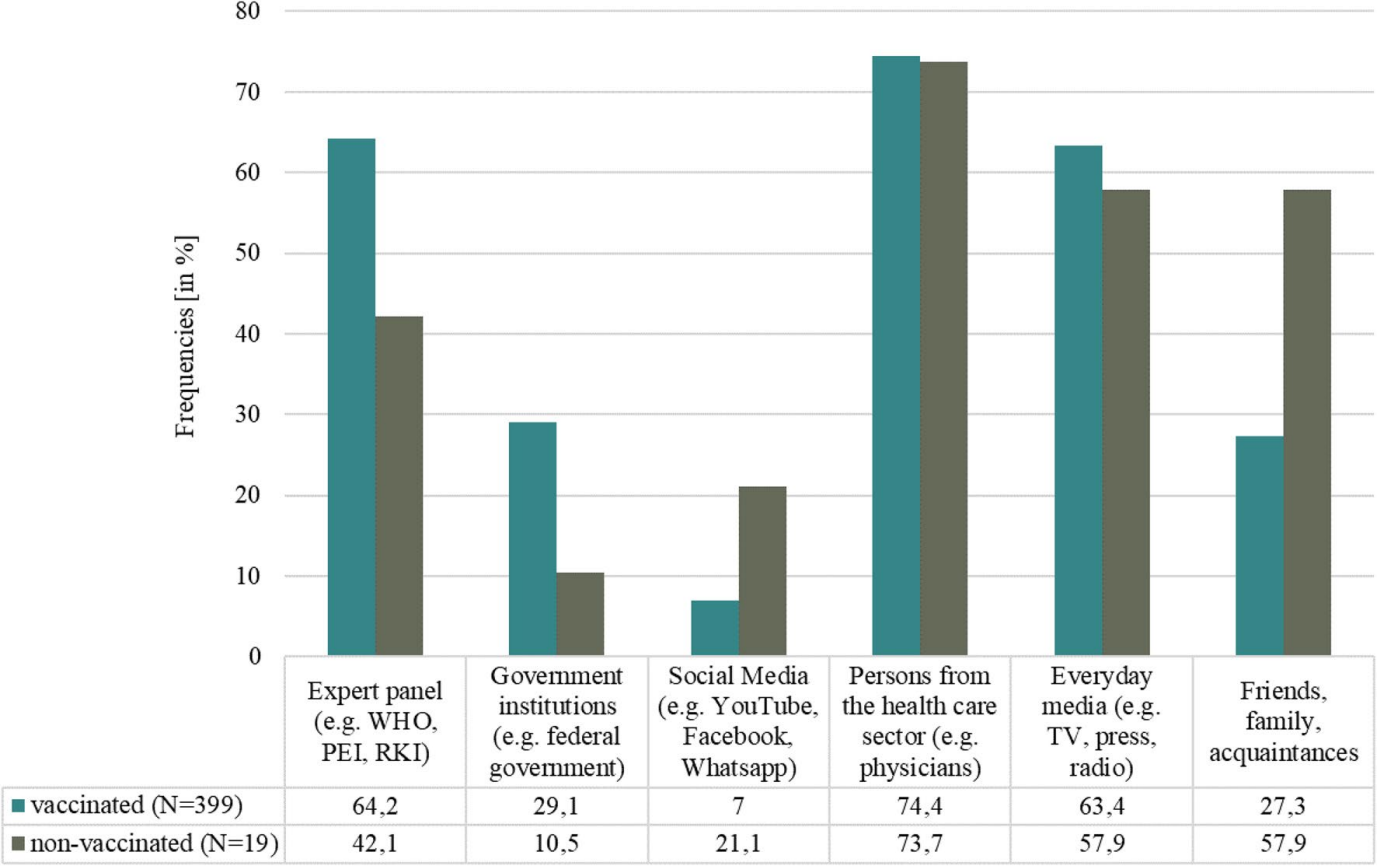
Percentage of sources used to obtain information on the COVID-19 vaccines^1^. *WHO* World Health Organization, *PEI* Paul-Ehrlich-Institut, *RKI* Robert-Koch-Institut, ^1^the possibility of multiple answers was given

The main sources of information on COVID-19 vaccination indicated by respondents were “persons from the health care sector” (75.0%) and both “expert panels” and “everyday media” (63.0%). Figure 4 shows the percentage of information sources used by vaccinated and nonvaccinated respondents, respectively.

**Fig. 5:**
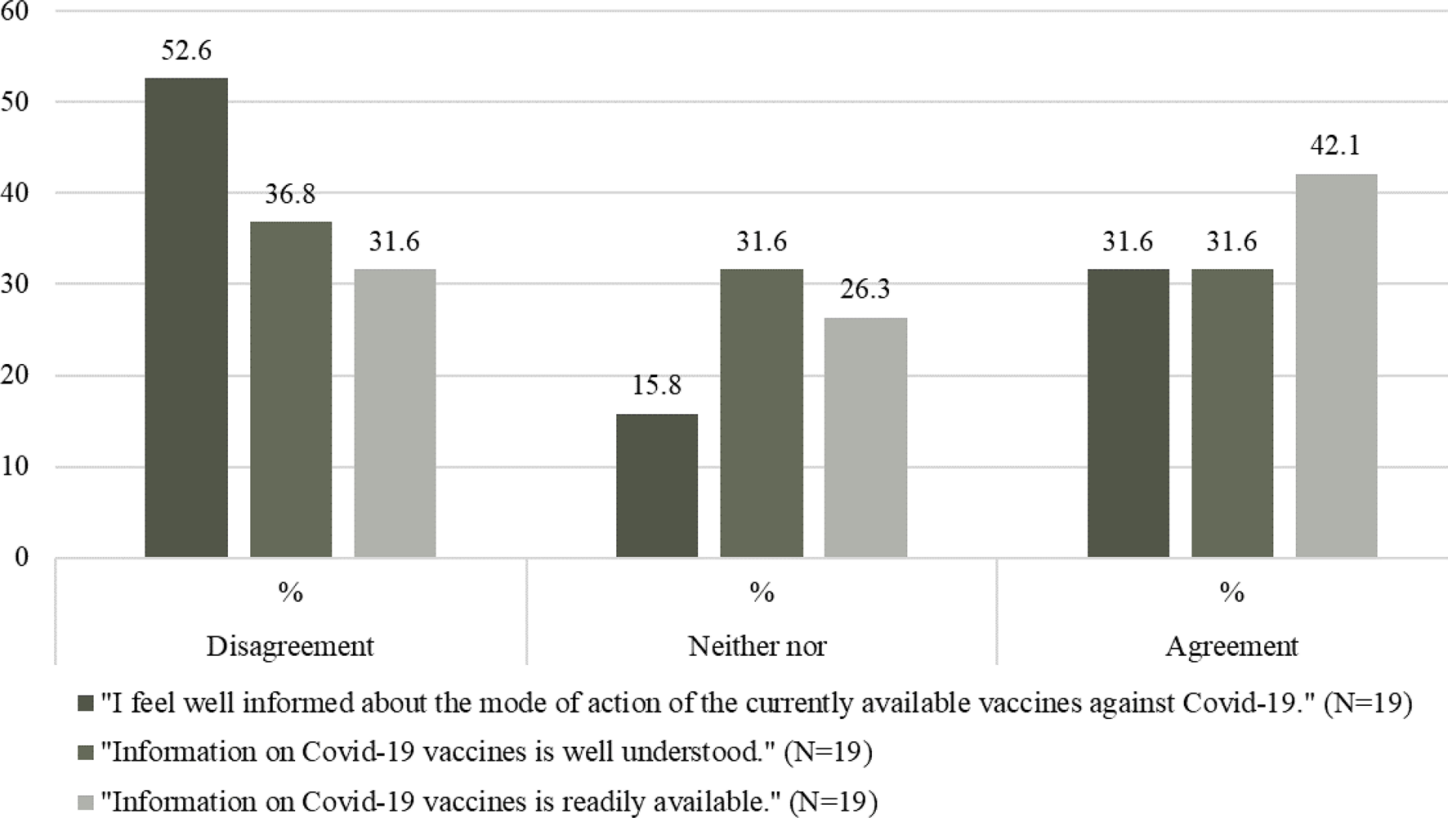
Percentage of level, comprehensibility and accessibility of information among nonvaccinated patients

**Fig. 6:**
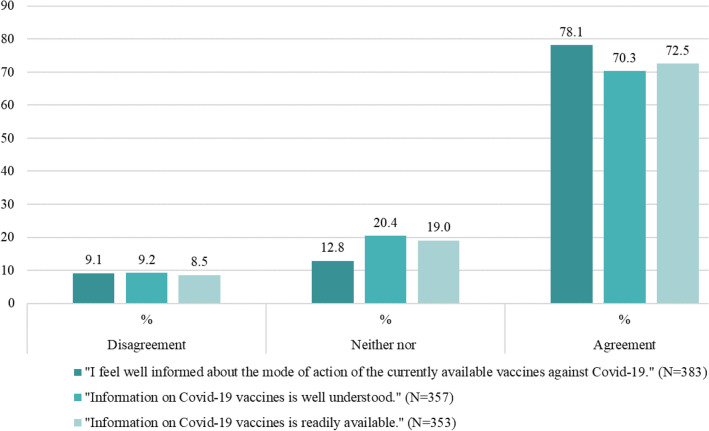
Percentage of level, comprehensibility and accessibility of information among vaccinated patients

In the total sample, the majority of patients (75.9%) feel well informed regarding COVID-19 vaccination, compared to those who disagree (11.2%) and those who are undecided (12.9%). In addition, the majority of respondents indicated that the information about COVID-19 vaccines is well understood (68.4%) and readily available (71.0%). Figures 5 and 6 show the percentage of agreement and disagreement, respectively, on the level, comprehensibility, and accessibility of information among nonvaccinated and vaccinated patients.

### Fear and anxiety symptomatology and health literacy

From 397 participants, 211 (49.6%) met criteria for minimal, 121 (28.5%) for mild, 47(11.1%) for moderate and 18 (4.2%) for severe anxiety symptomatology. From 418 participants, 411 (98.3%) did not reach a total score of ≥ 7 and thus may have problematic symptoms and probable dysfunctional thoughts about COVID-19. From 412 participants, 51 (12.0%) met criteria for inadequate, 142 (33.4%) for problematic and 219 (51.5%) for sufficient health literacy.

For means on fear and anxiety symptomatology and health literacy of the total sample, see Table 4. We found a significant negative correlation between vaccination status and obsessive thinking about COVID-19.

**Table 4:** Means, standard deviations, and correlations with confidence intervals

	Vaccination status			
Variables	*M*	*SD*	*r*	CI
Symptoms of anxiety (GAD-7)^1^	5.10	4.61	− 0.10	[− 0.19, 0.00]
Obsessive thinking toward COVID-19 (OCS)^2^	1.20	1.72	− 0.14**	[− 0.23, − 0.04]
Fear of COVID-19 (FCV-19S)^3^	13.47	5.74	− 0.08	[− 0.18, 0.01]
Health literacy (HLS-EU-Q16)^4^	12.35	3.03	0.02	[− 0.08, 0.12]

*M* and *SD* are used to represent mean and standard deviation, respectively. Values in square brackets indicate the 95% confidence interval for each correlation

^1^n/a = 28

^2^n/a = 7

^3^n/a = 6

^4^n/a = 13

** indicates *p* < 0.01

## Discussion

### Main findings and previous research

Our study presented data on COVID-19 vaccination and related psychosocial factors from (former) cancer patients across various cancer types. Our results demonstrate that the vast majority is vaccinated and “protection against severe course, disease consequences of infection” was the most mentioned reason among the vaccinated. This is in line with findings from a cross-sectional study among *N* = 999 French cancer patients, in which the majority (76.9%) reported “fear for their health” as the main reason for vaccination (Barrière et al 2021). With a current vaccination rate of 72.2% (BMG 2022) in the German population, the acceptance of COVID-19 vaccination is much lower than in our sample of (former) cancer patients.

We found that confidence in the protective effect of a vaccine is high in cancer patients with respect to a COVID-19 vaccine and vaccines in general. The most trusted sources of information on COVID-19 vaccines are expert panels, e.g., STIKO, RKI and treating physicians. This is consistent with a Germany-wide cross-sectional study in the general population with *N* = 1000 adults, in which physicians and expert panels such as the RKI and Bundeszentrale für gesundheitliche Aufklärung (BZgA) are trusted from a medium to high degree (Betsch et al 2020). Results from another study with *N* = 329 cancer patients from North Tunisia show that 77.5% had confidence in their physician (Mejri et al. 2022). This leaves physicians and other experts in the health care sector with a high responsibility in genuine and transparent communication, providing unbiased and clear information (Machingaidze and Wiysonge, 2021).

The vaccination readiness among nonvaccinated was low to very low with “fear of side effects” the most mentioned reason against a COVID-19 vaccine. This is in line with two studies from Tunisia and Italy. Of the respondents who chose not to be vaccinated, 33.1% and 48.1%, respectively, were concerned about side effects or adverse events associated with the vaccine (Mejri et al. 2022, Di Noia et al 2021). In contrast, the major study on the safety profile of Comirnaty® (BioNTech/Pfizer) with a total of *N* = 43,448 participants portrayed side effects characterized by short-term, mild-to-moderate pain at the injection site, fatigue, and headache. The incidence of serious adverse events was low and was similar in the vaccine and placebo groups (Polack et al 2020). However, understanding that vaccines can have side effects and that concerns about these effects exist, should be taken into account in communication between physicians and patients. Easy access to medical advice, if side effects occur, is critical for building trust and managing concerns before they trigger a level of anxiety that exacerbates negative experiences (Rief 2021).We found that information level was higher among vaccinated compared to nonvaccinated respondents, as well as the assessment of the comprehensibility of this information. Availability of information was rated well in our sample. Most common information sources were persons from the healthcare sector (e.g., physicians) among all respondents. Least common sources were Social Media (e.g., YouTube, Facebook, and WhatsApp) among the vaccinated and Government institutions (e.g., federal government) among the nonvaccinated patients. A survey from the Austrian Corona Panel Project showed similar results with only 19% of the nonvaccinated feel sufficiently well informed about vaccination, compared to those who have been vaccinated and those who are willing to be vaccinated at 55% and 40%, respectively (ACPP 2021). According to the WHO, the world is also fighting an “infodemic”, where facts are mixed with speculation, rumor and fear (Razai et al. 2021). To fill existing knowledge gaps, open dialogue and public involvement are essential instead of a passive, one-sided communication strategy (Mills et al 2020).

Our results showed no correlation between vaccination status and symptoms of anxiety in general and toward COVID-19. Overall, the majority of participants showed low levels of anxiety symptoms. The results demonstrated a significantly negative correlation between vaccination status and obsessive thoughts about COVID-19, with lower scores among vaccinated respondents. Our results are in contrast with results from a study by Head et al. (2020) with *N* = 3159 American adults highlighting a positive relationship between fear of COVID-19 and intention to get vaccinated.

There was no correlation between vaccination status and health literacy. Overall, more than half of the participants had sufficient health literacy. This is in line with a study from Germany, where COVID-19-related health literacy was measured within a sample of *N* = 1153 adults. 49.9% had “sufficient health literacy” with reference to COVID-19 (Paakkari and Okan 2020). In contrast to our results, a study by Montagni et al. (2021) found that the risk of being in “hesitant” was higher among individuals with a low health literacy score (OR = 1.44; 95% CI = [1.04;2.00]). In this sample of (former) cancer patients, health literacy could already be set higher from the outset. This might be because cancer patients are more intensively engaged with the possibilities of modern medicine and are more frequently and extensively educated about prevention strategies due to more intensive contact with physicians (Ludwig 2021).

### Strengths and limitations

Our study has several strengths. A wide range of patients were included regarding age (30 to 88 years) and cancer site. Second, patients had a wide range on time since diagnosis (up to 61 years) and thus we were able to include patients who were currently under treatment and those who were not. Lastly, we could gather data on a very topical and important issue among a highly relevant patient sample within our health care system.

However, this study has also limitation. One limitation lies in the small sample size of the subgroup of nonvaccinated respondents. Group comparisons should, therefore, be regarded as a trend. Second, we only surveyed cancer patients, who presented themselves at our counseling center. Those patients are already more integrated in the health care system, and therefore, the representativeness of the sample is limited. Conclusions only should be drawn for this special target group.

### Conclusions

In this study among cancer patients, the vast majority reported being vaccinated against COVID-19 with a positive attitude toward the vaccination. Considering obsessive thoughts about COVID-19 and vaccination status, we found first indications of a relationship. Further studies with a larger sample of nonvaccinated cancer patients should investigate whether this relationship can be verified, how and why those thoughts evolve and which role communication of responsible parties (e.g., politicians) and persons (e.g., physicians) takes. Targeted and tailored communication can help to further improve education, promote valid information, and build trust.

## Data Availability

The datasets generated during and/or analyzed during the current study are available from the corresponding author on reasonable request.
